# Structural insights into the non-inhibitory mechanism of the anti-EGFR EgB4 nanobody

**DOI:** 10.1186/s12860-022-00412-x

**Published:** 2022-03-01

**Authors:** Matthieu R. Zeronian, Sofia Doulkeridou, Paul M. P. van Bergen en Henegouwen, Bert J. C. Janssen

**Affiliations:** 1 Structural Biochemistry, Bijvoet Center for Biomolecular Research, Department of Chemistry, Faculty of Science, Utrecht University, Utrecht, The Netherlands; 2 Cell Biology, Neurobiology and Biophysics, Department of Biology, Faculty of Science, Utrecht University, Utrecht, The Netherlands; 3 Present address: Princess Máxima Center for Pediatric Oncology, Utrecht, The Netherlands

**Keywords:** Nanobody, Receptor, Structure, X-ray diffraction, EGFR-EGF

## Abstract

**Background:**

The epidermal growth factor receptor (EGFR) is involved in various developmental processes, and alterations of its extracellular segment are associated with several types of cancers, in particular glioblastoma multiforme (GBM). The EGFR extracellular region is therefore a primary target for therapeutic agents, such as monoclonal antibodies and variable domains of heavy chain antibodies (VHH), also called nanobodies. Nanobodies have been previously shown to bind to EGFR, and to inhibit ligand-mediated EGFR activation.

**Results:**

Here we present the X-ray crystal structures of the EgB4 nanobody, alone (to 1.48 Å resolution) and bound to the full extracellular EGFR-EGF complex in its active conformation (to 6.0 Å resolution). We show that EgB4 binds to a new epitope located on EGFR domains I and II, and we describe the molecular mechanism by which EgB4 plays a non-inhibitory role in EGFR signaling.

**Conclusion:**

This work provides the structural basis for the application of EgB4 as a tool for research, for targeted therapy, or as a biomarker to locate EGFR-associated tumors, all without affecting EGFR activation.

**Supplementary Information:**

The online version contains supplementary material available at 10.1186/s12860-022-00412-x.

## Background

The human epidermal growth factor receptor (HER) tyrosine kinase family is essential to cell growth, migration and differentiation, and is involved in the development of a variety of cancers [1–5]. Members of this family include EGFR, HER2, HER3 and HER4. Except for HER2, all members have been shown to bind to specific ligands [1], e.g. EGFR binds to epidermal growth factor (EGF) and transforming growth factor-α (TGF-α). EGFR was the first family member shown to be overexpressed in cancers [6] and it is therefore a primary target for anti-cancer therapies [7, 8]. New tools may help to further improve therapy and to characterize the role of EGFR in health and disease.

EGFR is a 170 kDa type I transmembrane receptor composed of an extracellular region characterized by four domains (I, II, III and IV), a transmembrane region, and an intracellular region composed of a kinase domain and a C-terminal tail. Ligand binding to the EGFR ectodomain is coupled to both homo- and heterodimerization [9], which is followed by conformational rearrangements of the transmembrane region and asymmetric dimerization of the intracellular kinase domains, one of which phosphorylates the other to initiate signaling [10–14]. In the resting state, the EGFR ectodomain samples between a tethered, inactive conformation, in which the domain II dimerization arm interacts with low affinity to domain IV, and an extended conformation, in which domain II has rotated 130° around domain III, therefore breaking the domain II – IV tether [15]. This open conformation creates a high affinity ligand binding pocket shared between domains I and III, which after ligand-binding exposes the dimerization arm for intermolecular interactions with another EGFR or with another member of the HER family [9, 15–17]. The ligand-bound EGFR extracellular region has some conformational plasticity in the dimer, most notably in the relative orientation of the two domains I and of the two membrane-proximal parts [18].

HER family members are expressed in all cell types and are critical to the embryogenesis of vertebrates [19]. In EGFR null mice, lethality was shown to be due to abnormalities in several organs including brain, lung, skin and gastrointestinal tract, and the renewal of stem cells [20, 21]. EGFR signaling remains also active in the mature central nervous system [22]. Besides its critical role in development and homeostasis, EGFR is involved in the initiation and maintenance of several types of solid tumors. Notably, the epidermal growth factor variant III (EGFRvIII) is found in ~ 40% of high-grade gliomas [23]. Other EGFR alterations include mutations in the kinase domain that are involved in ﻿non-small-cell lung cancers (NSCLCs), especially adenocarcinoma [24–26].

To treat EGFR-associated cancers, both small molecule inhibitors and monoclonal antibodies are increasingly used. However, the large size of monoclonal antibodies (~ 150 kDa) leads to reduced tumor penetration and slow distribution, and consequently to insufficient treatment efficacy [27–29]. The VHH domain of heavy-chain antibodies, also referred to as nanobody in its isolated form, is the domain responsible for antigen binding, and constitutes the smallest (~ 15 kDa) antigen-binding unit derived from natural sources [30]. Due to their small size and potential to bind to epitopes with a high affinity, nanobodies represent a valuable tool in cancer research, diagnostics and therapy [31, 32]. Nanobodies that bind to EGFR with a nanomolar affinity were selected from a *Llama glama*-derived phage display VHH library directed against EGFR [33–37], and structures of three inhibitory nanobodies (EgA1, 9G8 and 7D12) were solved in complex with the EGFR ectodomain in its inactive conformation [38]. All three nanobodies bind to domain III. The EgA1 and 9G8 nanobodies bind to a cleft formed between domains II and III, whereas the 7D12 interaction surface overlaps with the ligand binding site. These inhibitory nanobodies prevent EGFR from adopting an extended conformation that is required for ligand-mediated receptor activation. The EgB4 nanobody, selected from the VHH library directed against EGFR for its non-agonistic and non-antagonistic properties [34], was proposed to bind to EGFR domain I while not competing with EGF binding [34, 39], but no structural information is available on EgB4 or its interaction with EGFR. Here we report crystal structures of the EgB4 nanobody, alone (up to 1.48 Å resolution) and in complex with the full extracellular region of EGF-bound EGFR (up to 6.0 Å resolution). The structures explain the non-inhibitory binding of EgB4 to EGFR, the specificity of EgB4 for EGFR domains I and II, and indicate that EgB4 can bind both EGF-bound and unliganded EGFR. This work provides the structural basis for the use of EgB4 as a research tool, for targeting of drugs such as nanobody-drug conjugates, and as biomarker to monitor EGFR expression in tissues and tumor imaging, while not affecting EGFR function.

## Results

### Crystal structure of the EgB4 nanobody

**Fig. 1:**
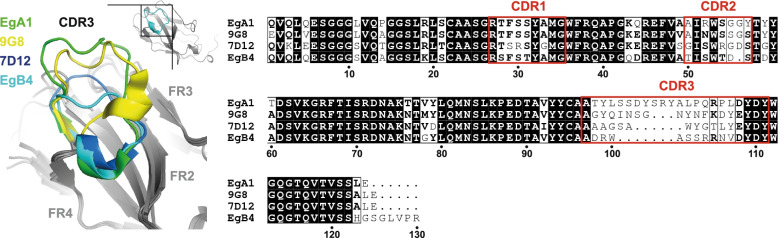
The CDR3 of EgB4 is substantially shorter than that of other anti-EGFR nanobodies. (Left) Structural alignment of four described anti-EGFR nanobodies showing the difference in CDR3 length. Inset shows the complete EgB4 nanobody. FR: framework region (grey). (Right) Corresponding sequence alignment with residues numbered according to the EgB4 sequence. A sequence alignment following the ImMunoGeneTics (IMGT) numbering is shown in Fig. S2

To investigate the structure of the EgB4 nanobody and its interaction with EGFR, we first determined a high-resolution structure of EgB4 (PDB: 7OM5). The EgB4 crystal diffracted to a maximum resolution of 1.48 Å (Table 1 and Fig. S1). The structure was solved by molecular replacement using the structure of the EgA1 nanobody (PDB: 4KRO) [38]. Model building and refinement led to a final model with *R*_work_/*R*_free_ of 0.177/0.205. Two EgB4 molecules are present in the asymmetric unit that align with a root mean square deviation (RMSD) of 0.17 Å. The framework regions of EgB4, defined as the conserved segments of nanobodies, align with that of a typical VHH [40] with a RMSD of 0.51 Å, whereas a RMSD of 3.9 Å is measured when aligning the complementarity determining regions (CDR). Notably, the CDR3 of EgB4 is relatively short compared to that of other nanobodies (Fig. 1) [38, 40].

**Table 1:** Data collection and refinement statistics

	EGFR-EgB4-EGF	EgB4
**Data collection**		
Space group	*P* 6_1_ 2 2	*P* 1 2_1_ 1
Cell dimensions		
*a*, *b*, *c* (Å)	307.61, 307.61, 135.14	38.54, 71.58, 53.20
*α, β, γ* (°)	90.0, 90.0, 120.0	90.0, 91.5, 90.0
Resolution (Å)	153.81 - 6.05 (7.15 - 6.05)	42.69 - 1.48 (1.50 - 1.48)
No. observed reflections	60,948 (6230)	162,212 (7549)
No. unique reflections	6321 (632)	47,214 (2189)
*R*_merge_	0.187 (1.995)	0.125 (1.681)
Mean I/σI	7.8 (1.6)	5.4 (1.1)
CC_1/2_	0.996 (0.548)	0.990 (0.277)
Spherical completeness (%)	65.3 (17.0)	98.0 (93.0)
Ellipsoidal completeness (%)	91.8 (63.9)	n/a
Ellipsoidal resolution limits (Å) [direction]	7.25 [a*]	n/a
	7.25 [b*]	
	6.02 [c*]	
Redundancy	9.6 (9.9)	3.4 (3.4)
**Refinement**		
Resolution (Å)	153.81 - 6.05	42.69 - 1.48
*R*_work_/*R*_free_ (%)	29.6 / 32.7	17.7 / 20.5
Average *B*-factors (Å^2^)		
Protein	534	17.4
Glycans/ions/ligands	591	15.7
Water	n/a	26.5
R.M.S. deviations		
Bond lengths (Å)	0.0021	0.0122
Bond angles (°)	0.57	1.68
Ramachandran (%)		
Favored	94.0	97.2
Allowed	5.9	2.8
Outliers	0.1	0
Molprobity score	1.59	1.21

Highest resolution shell in parentheses. *n/a* not applicable. *denotes reciprocal space

### EgB4 binds to domains I and II in the active dimeric EGFR-EGF complex

**Fig. 2:**
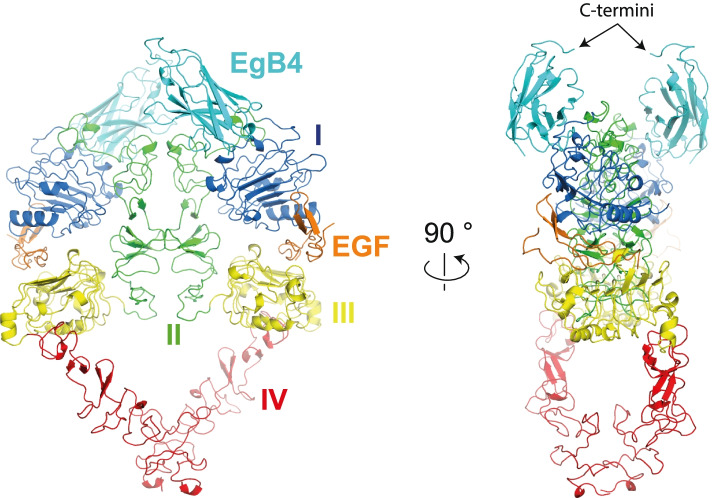
EgB4 nanobody binds to the active dimeric EGFR-EGF complex. The ternary complex, consisting of two EGFR, two EGF and two EgB4 molecules is shown in cartoon representation colored by domain. EgB4 binds mainly to EGFR domain I, with smaller contributions from EGFR domain II. EgB4 does not affect EGF binding, nor does it change the structure of the EGFR-EGF complex

To study the mechanism by which the EgB4 nanobody interacts with EGFR, we then determined the structure of the full ectodomain EGFR-EgB4-EGF ternary complex from a crystal that diffracted to a maximum resolution of 6.0 Å (PDB: 7OM4; Table 1 and Fig. S3). The structure was solved by molecular replacement, using one monomer of the EGFR-EGF complex (PDB: 3NJP, 3.3 Å resolution) [16] and one monomer of the EgB4 nanobody (described here, 1.48 Å resolution) as search models. Minimal model rebuilding, and refinement of the complex led to a final model with *R*_work_/*R*_free_ of 0.296/0.327 (see Methods). The structure shows a heart-shaped receptor-mediated dimer, on top of which EgB4 engages domains I and II, resulting in a physiological 2:2:2 complex (Fig. 2). The structure of the EGFR-EGF part of the EGFR-EgB4-EGF complex resembles closely the previously determined EGFR-EGF complex [16]. This indicates that the EGFR-EGF complex is in a physiologically active conformation when bound to EgB4, and that EgB4 binding does not induce large conformational changes in the EGF-bound EGFR.

**Fig. 3:**
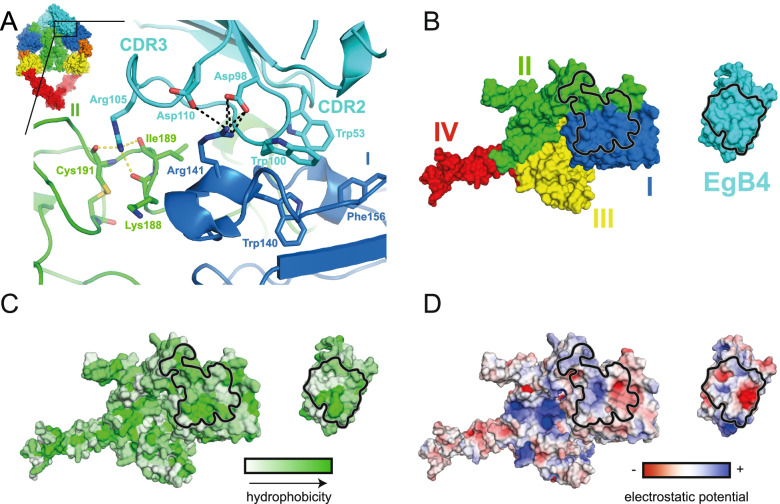
EgB4 nanobody interacts with EGFR domains I and II. **A** The CDR3 of EgB4 engages EGFR domains I and II while CDR2 of EgB4 binds to EGFR domain I. Residues involved in the interaction are shown in stick representation. Electrostatic and hydrogen bonding interactions are indicated by black and yellow dotted lines, respectively. Inset shows the EGFR-EgB4-EGF complex in surface representation. **B-D** Open book view of the EGFR-EgB4 complex with the interface delimited in black, colored by domains (**B**), hydrophobicity (**C**) and electrostatic potential (**D**)

Although several ligands and nanobodies were shown to bind to EGFR, EgB4 interacts with a hitherto unreported EGFR epitope. As shown in Fig. 3A, the CDR2 and CDR3 of EgB4 interact with the top of EGFR domain I, and CDR3 also interacts with residues at the domain I-II junction, together forming a buried surface area of 1403 Å^2^ (Fig. 3B). While sidechains are not resolved in the electron density of the 6 Å complex their location can be inferred from the correct positioning of the higher-resolution individual structures in the data. Specifically, a hydrophobic core is formed by the sidechains of Trp140 and Phe156 from the top of EGFR domain I, and by that of tryptophan residues in EgB4 CDR2 (Trp53) and CDR3 (Trp100) (Fig. 3A and C). Within the same region, the sidechain of EGFR Arg141 forms salt bridges with asparagine residues from EgB4 CDR3 (Asp98 and Asp110) (Fig. 3A and D). The CDR3 Arg105 sidechain forms additional hydrogen bonding interactions with the backbone carbonyl groups of Lys188, Ile189 and Cys191 at the EGFR domain I-II junction, extending the EGFR-EgB4 interface towards EGFR domain II (Fig. 3A). In the dimeric complex, although both EgB4 molecules bind at the top of EGFR domain I, they do not interact with each other (Fig. 2). Collectively, the data show that EgB4 binds to EGFR domains I and II of the physiological dimeric EGFR-EGF complex, therefore targeting a new epitope that could be used for research, diagnostic and therapeutic applications.

### EgB4 also binds unliganded EGFR

**Fig. 4:**
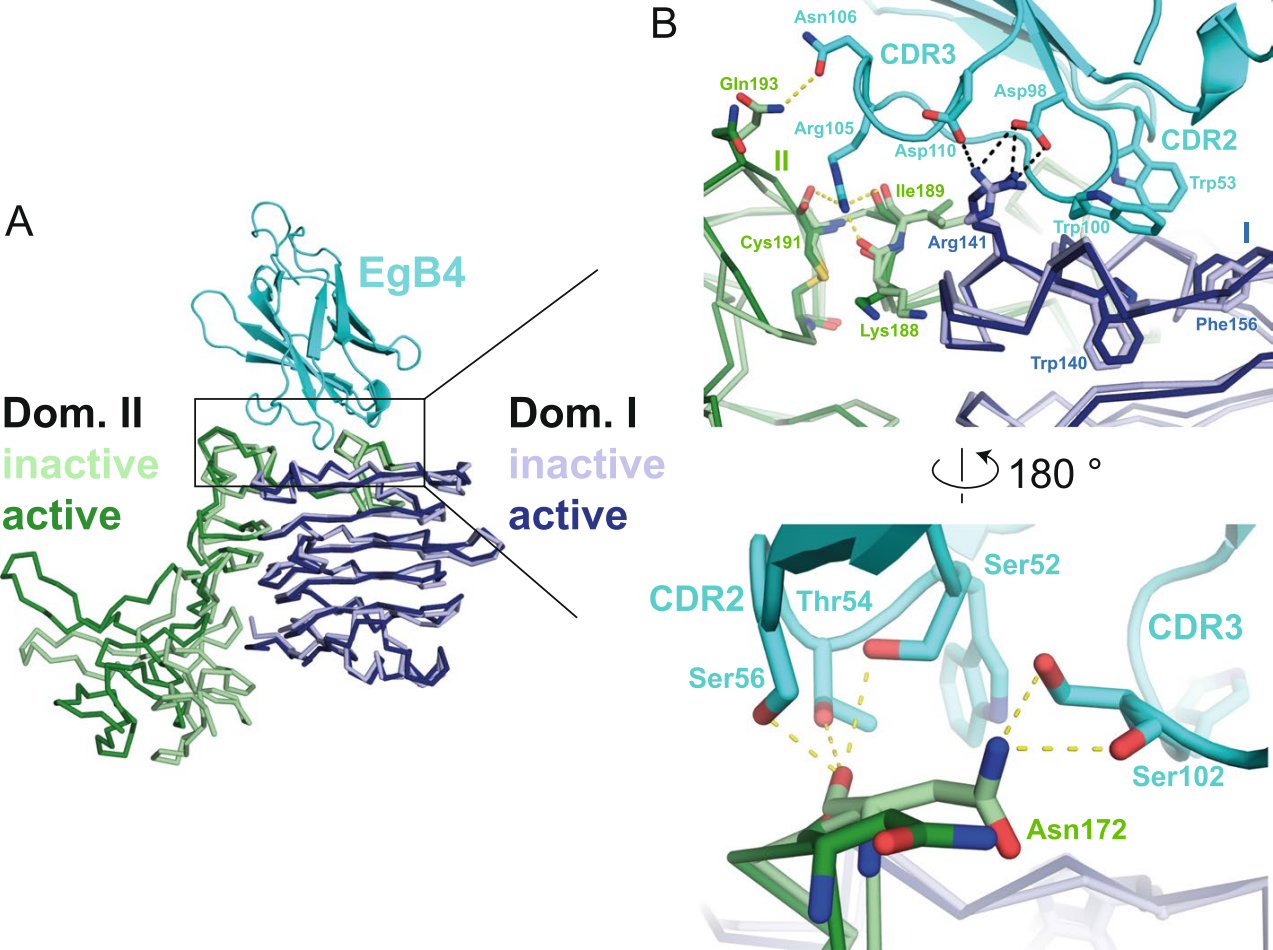
Modelling of the EgB4 – inactive EGFR complex. **A** Structural alignment of EGFR in the inactive, closed (light colors; unmodified from PDB: 3qwq) [41] and active EgB4-bound conformation (dark colors) in ribbon representation. The EgB4 — inactive EGFR complex is modelled by superposition based on the EgB4-EGFR interface from the crystal structure reported here. **B** Inactive, closed EGFR is also poised to interact with EgB4. Residues involved in the interaction are shown in stick representation. Electrostatic and hydrogen bonding interactions are indicated by black and yellow dotted lines, respectively. Potential steric clashes between EgB4 and residues Arg141 and Ile189 in the inactive EGFR are readily alleviated by selecting different rotamers

**Fig. 5:**
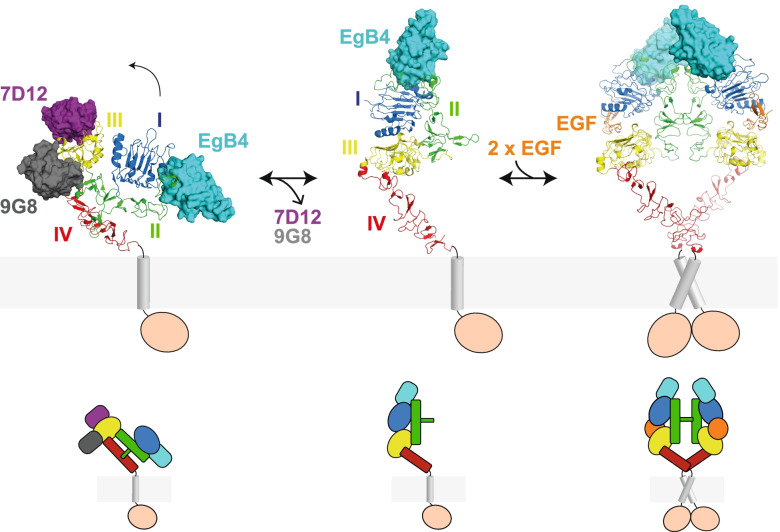
EgB4 nanobody does not prevent EGFR conformational rearrangement while 7D12 and 9G8/EgA1 nanobodies stabilize the inactive conformation. (Left) In the closed conformation, four nanobodies can bind to EGFR: EgB4, 7D12, 9G8 and EgA1 (PDB: 3qwq, 4krm, 4krp) [38, 41]. Here, EgB4 is modelled by superposition based on the EgB4-EGFR interface from the crystal structure reported here. (Center) In the extended monomeric conformation, only EgB4 may be able to bind to the unliganded EGFR (model based on HER2; PDB: 1n8z) [42]. (Right) Two EgB4 molecules can bind to the active dimeric EGFR. (Bottom) Corresponding schematic representations

The structure of the EgB4-binding site in the active EGFR resembles closely that of the inactive EGFR [41, 42], suggesting there is no interface rearrangement within EGFR in the transition from the inactive, closed conformation to the active ligand-bound conformation (Fig. 4A). Interactions that are formed between EgB4 and the active EGFR are therefore likely to also occur with the inactive, closed EGFR (Fig. 4B). In the EgB4 – inactive EGFR model, generated by straightforward superpositioning of the two EGFR forms based on the EgB4 binding site, additional hydrogen bonding interactions involving Asn172 from EGFR can be observed (Fig. 4B). It is possible that these additional interactions are also present in the active EGFR-EgB4-EGF complex, but not observed in the structure due to the low resolution of the underlying data. While experimentally we only determined the structure of the active EGFR-EgB4-EGF complex, the data shows that the inactive, closed EGFR conformation is also compatible with EgB4 engagement. Indeed, EgB4 binds to EGFR in the absence of EGF in size exclusion chromatography analysis (Fig. S4). Together, this suggests that EgB4 binds to the inactive EGFR and, unlike the 7D12, EgA1 and 9G8 nanobodies, allows the conformational change from the inactive, closed to the active EGFR conformation (Fig. 5).

## Discussion

EGFR is a widely studied receptor involved in various cellular processes, such as cell differentiation and migration, and its overexpression in cancers makes it an important therapeutical target [1–5]. EGFR-targeting drugs, including monoclonal antibodies (e.g. cetuximab), nanobodies (e.g. 7D12) or tyrosine kinase inhibitors (e.g. erlotinib) were designed to inhibit EGFR signaling by preventing conformational rearrangement of the receptor, competing with ligand binding, or blocking kinase activity [38, 43, 44]. Structures of the inhibitory nanobodies 7D12, EgA1 and 9G8 in complex with EGFR show that they all bind to EGFR in its inactive conformation, blocking conformational rearrangement of the receptor and therefore preventing formation of the extended active conformation [38]. All three nanobodies engage domain III, and while 7D12 interacts with the ligand binding region, EgA1 and 9G8 bind to a cleft created between domains II and III. Here we reveal the molecular details of EgB4 binding to EGFR by solving crystal structures of EgB4 alone, and in complex with EGF-bound EGFR, to provide structural information on the non-competing characteristics of EgB4. The data show that EgB4 binds to EGFR domains I and II through interactions with the variable regions CDR2 and CDR3. Most notably, a hydrophobic core constituted by tryptophan and phenylalanine residues at the top of EGFR domain I and tryptophan residues in CDR2 and CDR3, and electrostatic interactions between aspartic acid residues of EgB4 CDR3 and Arg141 on EGFR domain I, appear to be key to complex formation. The interaction is stabilized by additional hydrogen bonding interactions between the backbone carbonyl of Lys188, Ile189 and Cys191 on EGFR domain II and Arg105 in CDR3.

Nanobodies have various therapeutic applications. As an example, multimerization of the anti-EGFR 7D12 nanobody with other VHH domains has successfully led to the inhibition of tumor growth in vivo [35]. Moreover, biparatopic nanobodies have been used to induce oligomerization of the target receptor which leads to internalization of EGFR [45]. This principle can be used for intracellular targeting of drugs, increasing the efficacy of the targeted drug [46]. The EgB4 binding site on EGFR domains I and II is located relatively far from the previously described 7D12, 9G8 and EgA1 binding sites [38], preventing the straightforward design of a biparatopic molecule that includes EgB4 in combination with one of these nanobodies. However, fusion of EgB4 to another domain I-specific nanobody, called EgC9, is feasible and has resulted in a biparatopic nanobody construct that is inducing EGFR internalization [45]. Furthermore, the binding site for EgB4, located on EGFR domains I and II, could provide specificity on the type of EGFR variant that EgB4 can bind to. For example, EgB4 can probably bind to EGFRvII since that variant only lacks part of domain IV, but not to EGFRvIII that is truncated from most of its domains I and II. The use of EgB4 may thus help identify specific types of cancers that are characterized by the presence of EGFR or EGFRvII rather than EGFRvIII, and therefore provide useful insight into the type of cancer.

Only few anti-EGFR antibodies are known not to interfere with EGFR signaling. The rather unique property of the EgB4 nanobody to specifically bind to EGFR without interfering with its signaling, can be employed in basic research. Examples for application of EgB4 are the monitoring of EGFR trafficking in the plasma membrane [47], or the ligand-induced EGFR internalization into mammalian cells [45]. In addition, due to the small size, this nanobody is useful for the detection of molecular interactions by Förster resonance energy transfer (FRET), as exemplified by the detection of EGFR oligomerization in the plasma membrane [39, 48] and colocalization of EGFR with different lipids [34].

## Conclusions

The crystal structures of the EgB4 nanobody and of the ternary EGFR-EgB4-EGF complex reveal that EgB4 interacts with a hitherto unreported epitope located on EGFR domains I and II. Two complementarity-determining regions of EgB4, CDR2 and CDR3, of which CDR3 is substantially shorter than other EGFR-interacting nanobodies, are at the interface with EGFR. The interaction site is not changing in the conversion of EGFR from its closed, inactive conformation into the EGF-bound active state and reveals how EgB4 can interact with both the inactive and activated EGFR forms.

## Methods

### Expression and purification of EGFR, EgB4 and EGF

Codon-optimized DNA coding for human EGFR (UniProt Accession ID P00533) ectodomain (residues 1-621 of the mature protein) was purchased at GeneArt, subcloned in pUPE101.01 vector (U-Protein Express BV, C-terminal His6-tag) and transiently expressed in N-acetylglucoaminyltransferase I-deficient (GnTI-) Epstein-Barr virus nuclear antigen 1 (EBNA1)-expressing HEK293 cells growing in suspension (U-Protein Express BV). The medium was harvested 6 days after transfection and cells were spun down by 10 min of centrifugation at 1000x g. Protein was purified by Ni Sepharose excel (GE Healthcare) affinity chromatography, eluted with 500 mM imidazole in phosphate buffer saline (PBS) pH 7.4, and buffer-exchanged to PBS using the SnakeSkin™ Dialysis Tubing (Thermo Scientific). Size-exclusion chromatography (SEC) was performed on a Superdex200 10/300 increase column (GE Healthcare) equilibrated in SEC buffer (20 mM HEPES pH 7.4, 150 mM NaCl). Protein purity was evaluated by Coomassie-stained sodium dodecyl sulphate-polyacrylamide gel electrophoresis (SDS-PAGE). Protein was concentrated and stored at − 80 °C.

Codon-optimized DNA coding for EgB4 was purchased at Integrated DNA Technologies (IDT BVA), cloned into a customized pHEN6 vector with a pelB sequence for expression in the bacterial periplasm and a thrombin cleavage site followed by a C-terminal His6-tag. Protein was expressed under IPTG induction in BL21-CodonPlus (DE3)-RIL *E. coli* bacteria cultured in Terrific Broth medium in a New Brunswick™ BioFlo®/CelliGen® 115 bioreactor (pH 7 ± 0.1 and dissolved oxygen 70%). The periplasm was extracted from the harvested bacteria via two rounds of freeze-thaw (− 20 °C) and was collected in PBS. The nanobody was purified from the isolated periplasm by Ni Sepharose™ High Performance chromatography, eluted in 500 mM imidazole in phosphate buffer saline (PBS) pH 7.4, and buffer-exchanged to PBS using a HiTrap™ Desalting column (GE Healthcare). The C-terminal His6-tag was removed by thrombin cleavage and SEC was performed on a Superdex75 10/300 increase column (GE Healthcare) equilibrated in SEC buffer. Protein purity was evaluated by Coomassie-stained SDS-PAGE. Protein was concentrated, and stored at − 80 °C.

Human EGF (UniProt Accession ID P01133) was bought from Sino Biological Inc., reconstituted in Milli-Q® water, and purified by SEC on a Superdex75 10/300 increase column (GE Healthcare) equilibrated in SEC buffer. Protein purity was evaluated by Coomassie-stained SDS-PAGE. Protein was concentrated and stored at − 80 °C.

### Crystallization and data collection

The EGFR-EgB4-EGF complex crystal grew by sitting-drop vapour diffusion at 20 °C, by mixing 150 nL of 10 mg/mL protein solution containing EGFR:EgB4:EGF in 1:1.1:1.1 M ratio, respectively, with 150 nL of reservoir solution containing 0.1 M LiSO_4_, 0.1 M glycine pH 10.5, 1.1 M sodium dihydrogen phosphate and 0.72 M dipotassium hydrogen phosphate. The crystal was harvested and flash-cooled in liquid nitrogen in presence of reservoir solution supplemented with 20% glycerol. The dataset was collected at 100 K at the Diamond Light Source (DLS) beamline I24 (λ = 0.9686 Å).

The EgB4 crystal grew by sitting-drop vapour diffusion at 20 °C, by mixing 150 nL of protein solution at 22.4 mg/mL with 150 nL of reservoir solution containing 0.05 M zinc acetate and 20% w/v PEG3350. The crystal was harvested and flash-cooled in liquid nitrogen in presence of reservoir solution supplemented with 25% glycerol. The dataset was collected at 100 K at the DLS beamline I24 (λ = 0.9688 Å).

### Structure solution and refinement

The EGFR-EgB4-EGF complex data, to 6 Å resolution, was processed in the autoPROC pipeline [49], and additional anisotropic correction was done using the STARANISO server [50]. Unobserved and unobservable reflections that lie outside the diffraction cut-off surface were removed from the merged data file. The structure was solved by molecular replacement in PHASER [51], using one copy of the higher-resolution EGFR-EGF complex (PDB: 3NJP [16]) and one copy of the high-resolution EgB4 nanobody (described here). One copy of each molecule is present in the asymmetric unit. The structure solution and subsequent refinement were aided by the availability of the higher resolution structure of EGFR-EGF, to 3.3 Å resolution [16], and EgB4, to 1.48 Å resolution. To minimize overfitting the low-resolution refinement was done in REFMAC and PHENIX using only TLS parameters to model the B-factors (one group per EGFR domain, one group for EgB4 and one group for EGF) and using jelly-body and tight geometry restraints [52–54]. Minimum manual rebuilding was done in COOT to correct Ramachandran outliers [55]. MOLPROBITY [56] was used for validation. The final model has a *R*_work_/*R*_free_ of 0.296/0.327 and was deposited to the Protein Data Bank (PDB) under the accession code 7OM4.

The EgB4 data was processed in the XIA2 pipeline [57]. The structure was solved by molecular replacement in PHASER [51], using one copy of the EgA1 nanobody as search model. Two copies of EgB4 are present in the asymmetric unit. Refinement was done in REFMAC [52], and MOLPROBITY [56] was used for validation. The final model has a *R*_work_/*R*_free_ of 0.177/0.205 and was deposited to the Protein Data Bank under the accession code 7OM5. Sequence alignment was done in Clustal Omega [58] and represented with ESPript [59]. Structure alignments were made in Pymol using the “align” command, and figures were made using Pymol [60].

### Isolation of the EGFR-EgB4 complex in solution

The EGFR-EgB4 complex was incubated in a 1:1.1 M ratio for 1 h at 20 °C. SEC was performed on a Superdex200 10/300 increase column (GE Healthcare) equilibrated in SEC buffer. Fractions from P1 and P2 were analyzed by Coomassie-stained SDS-PAGE.

## Supplementary Information

**Additional file 1.**

## Data Availability

Coordinates and structure factors for the EgB4 nanobody and the EGFR-EgB4-EGF complex have been deposited in the Protein Data Bank (PDB) (www.wwpdb.org) with accession codes 7OM5 and 7OM4, respectively. Other data generated during this study are included in this article or available from the corresponding author on reasonable request.
