# Supplementary material for: Structural insights into the non-inhibitory mechanism of the anti-EGFR EgB4 nanobody
Source: BMC Mol Cell Biol. 2022 Mar 1;23:12. doi: 10.1186/s12860-022-00412-x (PMC8887186; doi:10.1186/s12860-022-00412-x)
Supplement: Supplementary file 1 — Additional file 1. [file 12860_2022_412_MOESM1_ESM.pdf]

Supplementary information

Structural insights into the non-inhibitory mechanism of the anti-EGFR EgB4 nanobody

Matthieu R. Zeronian,[1] Sofia Doulkeridou,[2,3] Paul M.P. van Bergen en Henegouwen[2] and Bert J.C. Janssen[1]*

[1]Structural Biochemistry, Bijvoet Center for Biomolecular Research, Department of Chemistry, Faculty of Science, Utrecht University, Utrecht, The Netherlands

[2]Cell Biology, Neurobiology and Biophysics, Department of Biology, Faculty of Science, Utrecht University, Utrecht, The Netherlands

[3]Present address: Princess Máxima Center for pediatric oncology, Utrecht, The Netherlands

*Corresponding author, b.j.c.janssen@uu.nl

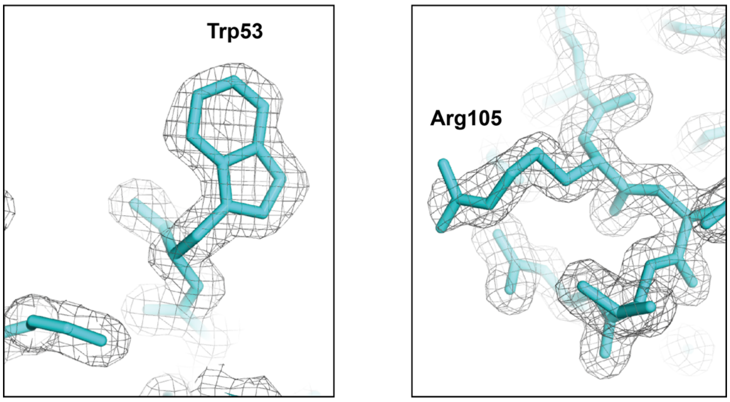

Figure S1. 2mF$_{obs}$-DF$_{calc}$ map of residue sidechains at 1 σ level in the individual EgB4 to 1.48 Å. The residues shown are involved in the interaction with EGFR in the EGFR-EgB4-EGF complex.

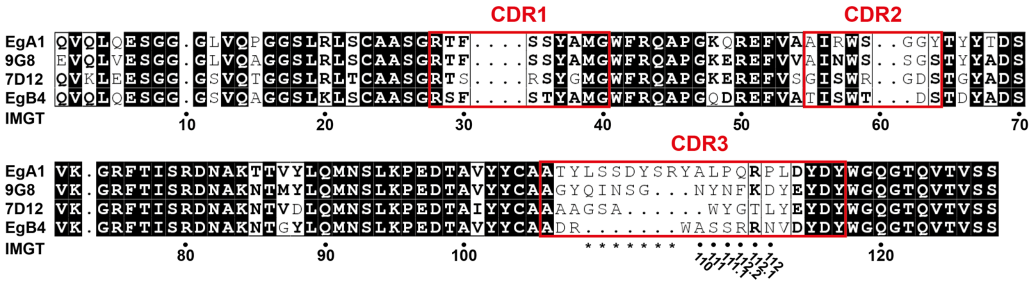

Figure S2. Sequence alignment of anti-EGFR nanobodies with IMGT numbering. Residues are numbered according to the EgB4 sequence. Insertions in non-EgB4 sequences are represented by an asterisk.

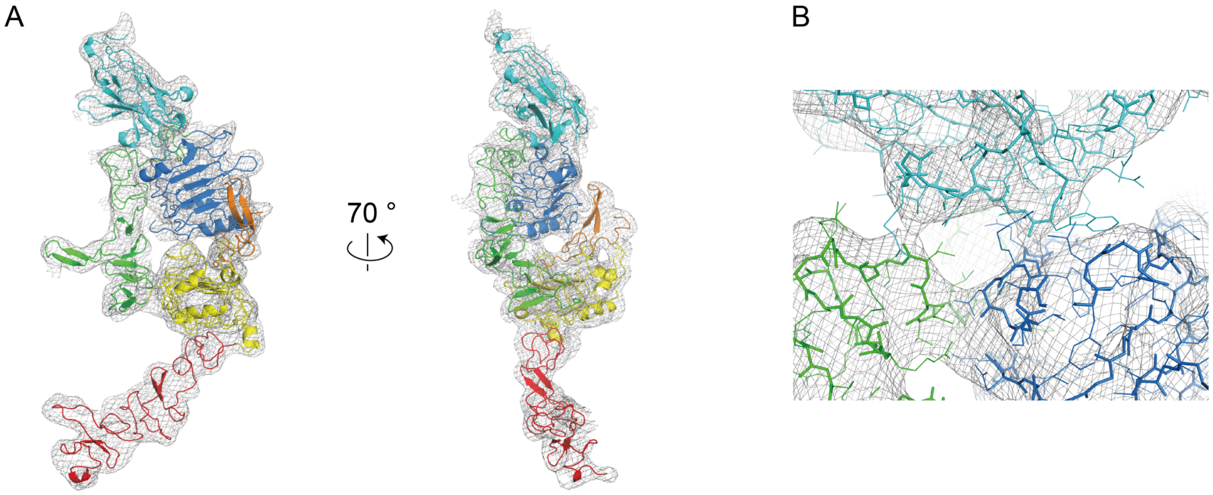

Figure S3. Electron density maps of the EGFR-EgB4-EGF complex. A) 2mF_{obs}-DF_{calc} map of the EGFR-EgB4-EGF complex at 1 σ level and carved at 3 Å around the model obtained after molecular replacement and before refinement, indicating the correct placement of all the molecules. B) 2mF_{obs}-DF_{calc} map of the EGFR-EgB4 interface at 1 σ level after minimal model refinement.

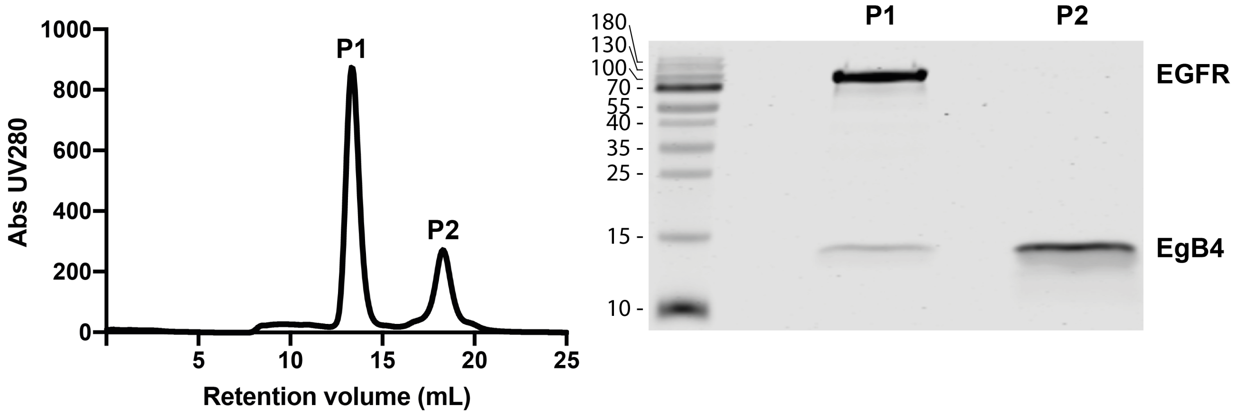

Figure S4. EgB4 binds to unliganded EGFR in solution. Preincubated EgB4 and EGFR coeluted in peak (P) 1 in SEC, as seen from the chromatogram (left) and the corresponding Coomassie-stained SDS-PAGE (right). P2 represents the excess EgB4.
